# Oligo-recurrence predicts favorable prognosis of brain-only oligometastases in patients with non-small cell lung cancer treated with stereotactic radiosurgery or stereotactic radiotherapy: a multi-institutional study of 61 subjects

**DOI:** 10.1186/s12885-016-2680-8

**Published:** 2016-08-19

**Authors:** Yuzuru Niibe, Tetsuo Nishimura, Tetsuya Inoue, Katsuyuki Karasawa, Yoshiyuki Shioyama, Keiichi Jingu, Hiroki Shirato

**Affiliations:** 1 Department of Radiology and Radiation Oncology, Kitasato University School of Medicine, 1-15-1, Kitasato, Minami-ku, Sagamihara, Kanagawa 252-0374 Japan; 2 Division of Radiation Oncology, Shizuoka Cancer Center, 1007, Monagakubo, Nagaizumi-cho, Sunto-gun, Shizuoka 411-8777 Japan; 3 Department of Radiation Medicine, Hokkaido University Graduate School of Medicine, Kita 15-jo, Nishi 7-chome, Kita-ku, Sapporo, Hokkaido 060-8638 Japan; 4 Department of Radiation Oncology, Tokyo Metropolitan Cancer and Infectious Diseases Center Komagome Hospital, 3-18-22, Honkomagome, Bunkyo-ku, Tokyo 113-8677 Japan; 5 Department of Clinical Radiology, Graduate School of Medical Sciences, Kyushu University, 3-1-1, Maidashi, Higashi-ku, Fukuoka 812-8582 Japan; 6 Ion Beam Therapy Center, SAGA-HIMAT Foundation, 415, Harukoga-cho, Tosu, Saga 841-0071 Japan; 7 Department of Radiation Oncology, Tohoku University Graduate School of Medicine, 2-1, Seiryo-machi, Aoba-ku, Sendai, Miyagi 980-8575 Japan; 8 Department of Radiology, Toho University Omori Medical Center, 6-11-1, Omori-nishi, Ota-ku, Tokyo 143-8541 Japan

**Keywords:** Oligometastases, Oligo-recurrence, Non-small cell lung cancer (NSCLC), Stereotactic radiosurgery (SRS), Stereotactic radiotherapy (SRT)

## Abstract

**Background:**

To investigate the prognostic value of oligo-recurrence in patients with brain-only oligometastases of non-small cell lung cancer (NSCLC) treated with stereotactic radiosurgery (SRS) or stereotactic radiotherapy (SRT).

**Methods:**

Patients treated with SRS or SRT for brain-only NSCLC oligometastases in 6 high-volume institutions in Japan between 1996 and 2008 were reviewed. Eligible patients met 1), 2), and 4) or 1), 3), and 4) of the following: 1) NSCLC with 1 to 4 brain metastases on magnetic resonance imaging (MRI) treated with SRS or SRT; 2) control of the primary lesions (thorax) at the time of SRS or SRT for brain metastases (patients meeting this criterion formed the oligo-recurrence group); 3) with SRS or SRT for brain metastases, concomitant treatment for active primary lesions (thorax) with curative surgery or curative stereotactic body radiotherapy (SBRT), or curative chemoradiotherapy (sync-oligometastases group); and 4) Karnofsky performance status (KPS) ≥70.

**Results:**

The median overall survival (OS) of all 61 patients was 26 months (95 % CI: 17.5–34.5 months). The 2-year and 5-year overall survival rates were 60.7 and 15.7 %, respectively. Stratified by oligostatus, the sync-oligometastases group achieved a median OS of 18 months (95 % CI: 14.8–21.1 months) and a 5-year OS of 0 %, while the oligo-recurrence group achieved a median OS of 41 months (95 % CI: 27.8–54.2 months) and a 5-year OS of 18.6 %. On multivariate analysis, oligo-recurrence was the only significant independent factor related to a favorable prognosis (hazard ratio: 0.253 (95 % CI: 0.082–0.043) (*p* = 0.025).

**Conclusions:**

The presence of oligo-recurrence can predict a favorable prognosis of brain-only oligometastases in patients with NSCLC treated with SRS or SRT.

## Background

Stage IV or recurrent stage IV patients have the shortest overall survival. In non-small cell lung cancer (NSCLC), the median overall survival is only 8 months, [1].

However, recent advances in molecular targeted drug have not only improved the QOL of NSCLC patients, but given them hope for survival. For example, patients with EGFR mutant adenocarcinoma lung cancer (a type of NSCLC) treated with EGFR-TKI have been reported to achieve long-term survival while maintaining good performance status [2]. EML4-ALK NSCLC patients (adenocarcinoma only) treated with ALK-inhibitor have also been shown to achieve long-term median survival [2]. However, these findings were limited to patients with driver oncogene mutations and driver-targeted therapy for adenocarcinoma only. The results for squamous cell carcinoma, large cell carcinoma, and other types, as well as for adenocarcinoma not having driver oncogene mutations, are much worse, as mentioned.

Furthermore, the personalized therapies for NSCLC are no longer limited to molecular targeted drugs. Indeed, there is a broad array of options beyond the molecular approach. Hellman, Wechselbaum, and Niibe were the first to propose the concepts of oligometastases and oligo-recurrence [3–5].

Oligometastases is defined as cases with 1 to 5 metastatic lesions, mostly with an active primary lesion, which are treated with local therapy (metastatic lesions) and can achieve long-term survival [3].

Oligo-recurrence [4–7], on the other hand, is defined as cases having 1–5 metastatic or recurrent lesions with controlled primary lesions, which are treated by local therapy such as surgery, stereotactic radiosurgery (SRS), stereotactic body radiotherapy (SBRT), radiofrequency ablation (RFA), and so on. These local therapies are strong and minimally invasive. Thus, patients with oligo-recurrence are treated for all gross tumors to maintain QOL and can achieve long-term survival or, in some cases, even cure, independent of their driver oncogene status. Thus, Palma and Wechselbaum et al. emphasized the importance of distinguishing between oligo-recurrence and oligometastases precisely because oligo-recurrence carries such a hopeful prognosis [8].

The current study investigates the importance of oligo-recurrence comparing with sync-oligometastases in patients with brain-only NSCLC oligometastases. Non-small lung cancer (NSCLC) patients with brain metastases is not rare. However, sync-oligometastases (Brain-only metastases NSCLC with active primary lesions were treated with local therapy for primary lesions and SRS or SRT for brain metastases) were very rare and as far as we know, this is first clinical demonstration of treatment outcomes of sync-oligometastases of NSCLC with brain-only metastases. Furthermore, the current study also investigated an analysis of the prognostic value of oligo-recurrence in comparison with other previously reported factors.

## Methods

### Patients

The patients in the current study were treated with SRS or SRT for brain-only NSCLC oligometastases at six university hospitals or major cancer centers (Kitasato University Hospital, Hokkaido University Hospital, Shizuoka Cancer Center, Cancer and Infectious Diseases Tokyo Metropolitan Komagome Hospital, Kyushu University Hospital, and Tohoku University Hospital) between 1996 and 2008. All institutional review boards approved this study (Ethics Committee of Kitasato University School of Medicine (B), Instittutional Review Board of Hokkaido university Hospital for Clinical Research, Ethics Committee of Shizuoka Cancer Center, Ethical Committee of Tokyo Metropolitan Komagome Hospital, Kyushu University Institutional Review Board for Clinical Research, Ethics Committee of Tohoku University Graduate School of Medicine). This study is retrospective. Thus, informed consent of all patients could not be acquired. Then, all institutions engaged in this study announced this study on the web and/or posters at the out-patients clinics at each hospital. If targeted patients would not like to engage in this study, they would convey their refusal to the researchers by face to face, telephone or e-mail. However, no patients proposed not to engage in this study.

Of the following criteria, eligible patients met 1), 2), and 4) or 1), 3), and 4): 1) NSCLC with 1 to 4 brain metastases detected by magnetic resonance imaging (MRI) treated with SRS or SRT; 2) control of the primary lesions (thorax) at the time of SRS or SRT for brain metastases (patients meeting this criterion formed the oligo-recurrence group); 3) with SRS or SRT for brain metastases, concomitant treatment for active primary lesions (thorax) with curative surgery or curative SBRT or curative chemoradiotherapy for primary lesions (sync-oligometastases group, where “sync” indicates “synchronous”) [7, 9]; 4) Karnofsky performance status (KPS) ≥70. The exclusion criteria were: 1) NSCLC with five or more brain metastases detected by MRI; and 2) NSCLC with 1 to 4 brain metastases for which surgery was previously performed.

・We compared the characteristics of oligo-recurrence group and sync-oligometastases.

There were no statistically differences among these two groups as following.

### SRS and SRT treatments

Head rings were attached to the NSCLC patients and fixed to the linear accelerator during SRS. The SRS dose prescription was given at the tumor peripheral margin (GTV + 1 mm = CTV, CTV + 1 mm = PTV) (PTV peripheral dose).

NSCLC patients were treated with SRT while fixed to the linear accelerator by head and face shells. The SRT dose prescription was given at the tumor peripheral margin (GTV + 1 mm = CTV, CTV + 2 mm = PTV) (PTV peripheral dose). SRT was delivered in 4 to 5 fractions.

### Treatments for thoracic lesions

Because patients in the oligo-recurrence group had controlled primary lesions, no further treatments of thoracic lesions were performed in this group until thoracic relapse. However, the sync-oligometastases group had active thoracic lesions. Therefore, in this group, the thoracic lesions were treated with curative surgery, SBRT (cT1N0M1BRA) and concurrent chemoradiotherapy, or with curative radiation therapy alone. SBRT was mainly performed using 48 Gy/4 fractions (isocenter dose) to the small primary lung cancer. Concurrent chemoradiotherapy and curative radiation therapy alone were mainly performed using 60 Gy/30 fractions (in all cases, the spinal cord dose was under 40 Gy).

In general, the treatment strategy was to attempt to target all gross malignant tumors.

### Statistical analyses

Overall survival (OS), relapse-free survival (RFS), local control of brain metastases (Brain-LC), cranial local control (Cranial–LC), and local control of thoracic lesions (Thoracic-LC) were calculated by the Kaplan-Meier method.

Overall survival was calculated from the date of the start of SRS or SRT for brain metastases, and an event was defined as any death. Relapse-free survival (RFS) was also calculated from the date of the start of SRS or SRT for brain metastases, and the events were defined as any site of relapse and any death. Local control of brain metastases (Brain-LC) was calculated from the date of the start of SRS or SRT for brain metastases, and the event was defined as more than 25 % regrowth (diameter) of brain metastases treated with SRS or SRT. Thus, the emergence of new lesions in the brain was not counted as an event when calculating Brain-LC. Cranial local control (Cranial-LC) was also calculated from the date of the start of SRS or SRT for brain metastases, and the events were any type of cranial relapse, including at the sites of SRS or SRT treatment, as well as the emergence of new lesions in the brain regions not treated with SRS or SRT. Local control for thoracic lesions (Thoracic-LC) was calculated from the date of thoracic lesion control by surgery or SBRT and concurrent chemoradiotherapy. These dates were defined as the surgery date, and the initiation date of SBRT or concurrent chemoradiotherapy. An event was defined as any type of intrathoracic relapse.

Univariate analysis of prognostic factors was performed by the log-rank test for OS, Cranial-LC, Brain-LC, and Thoracic-LC. The cut-off level of significance was defined as *p* < 0.05.

For OS, Cranial-LC, Brain-LC, and Thoracic-LC, multivariate analyses were also performed using Cox proportional hazards models. The factors used in these analyses were defined as those that were significant (*p* < 0.05) or showed a nonsignificant trend toward significance (*p* < 0.25) on univariate analysis and clinically important factors such as RPA class, which was previously reported to be a prognostic factor for brain metastasis and is widely used for classification.

## Results

A total of 61 patients in 6 major hospitals were registered. The detailed characteristics of the patients are listed in Table 1. Furthermore, the current study compared the background of oligo-recurrence and sync-oligometastases. There were no statistically differences among these two groups, indicating in Table 2.

**Table 1:** Patients’ characteristics

Characteristic	No.		Percent
Age, median (range), y		64 (22–86)	
<65	30		49
≥65	31		51
Sex			
Male	30		49
Female	31		51
No. of lesions			
1–2	54		89
3–5	7		11
Oligostatus			
Sync-oligometastases (primary active)	11		18
Oligo-recurrence (primary controlled)	50		82
Histological status			
Sguamous cell carcinoma	6		10
Adenocarcinoma	48		79
Others	7		11
KPS score			
70–80	5		8
90–100	56		92
Interval to initial brain recurrence, months			
<12	29		48
≥12	32		52
No. of brain metastases			
Single	42		69
2–4	19		31
RPA			
Class I (aged <65 years; no active extracranial diseases)	26		43
class II (aged ≥65 years; active extracranial diseases)	35		57
GPA			
Score 0–1.0 (scoring based on Age, KPS, ECM, No. of BM)	0		0
Score 1.5–2.0	5		8
Score 2.5–3.0	45		74
Score 3.5–4.0	11		18
Neurologic function^a^			
Grade 0	40		66
Grade 1	12		20
Grade 2	9		14
Grade 3	0		0
Grade 4	0		0
Maximum diameter of brain metastases, cm			
Median (range)		1.2 (0.2–6.0)	
Treatment method for brain tumor			
SRS	45		74
SRT	16		26
Dose at the brain tumor margin, Gy			
Median (range)		25 (10–36)	
WBRT	9		15
Thoracic stage^b^			
I-II	27		44
III	34		56
Treatment method for thoracic lesions			
Surgery	43		70
Radiation therapy	18		30
Chemotherapy	19		31

Abbreviations: *KPS* Karnofsky performance status, *RPA* recursive partition analysis, *GPA* graded prognostic assessment, *SRS* stereotactic radiosurgery, *SRT* stereotactic radiotherapy, *WBRT* whole brain radiation therapy

^a^Neurologic function, grade 0 as no symptoms; grade 1 as minor symptoms, fully active without assistance; grade 2 as moderate symptoms, fully active but reguires assistance; grade 3 as moderate symptoms, less than fully active, reguires assistance; grade 4 as severe symptoms, totally inactive

^b^Thoracic stage classified according to the TNM classification of malignant tumors version 6 (UICC, Union for International Cancer Control version 6 edition) not evaluating M stage

**Table 2:** Characteristics of Patients. Comparing oligo-recurrence with sync-oligometastases

Characteristic	Total No.	Oligo-recurrence group No	Sync-oligometastases group No	*p* value
Age,				
<65	30	24	6	*NS*
>65	31	26	5	
Sex				
Male	30			*NS*
Female	31			
No. of metastatic/recurrent lesions				
1–2	54	45	9	*NS*
3–5	7	5	2	
Histological status				
squamous cell carcinoma	6	5	1	*NS*
adenocarcinoma	48	40	8	
others	7	5	2	
KPS score				
70–80	5	5	0	
90–100	56	45	11	
interval to brain recurrence, mo				
<12	29	24	5	*NS*
>12	32	26	6	
Neurologic function ^a^				
grade 0	40	31	7	*NS*
grade 1	12	10	2	
grade2	9	9	0	
grade3	0	0	0	
grade4	0	0	0	
Treatment method for brain tumor				
SRS	45	37	8	*NS*
SRT	16	13	3	
WBRT	9	7	2	*NS*
Thoracic stage ^b^				
I–II	27	21	6	*NS*
III	34	29	5	
Treatment method for thoracic lesions				
Surgery	43	37	6	*NS*
Radiation therapy	18	13	5	
Chemotherapy	19	12	7	*NS*

Abbreviations: *KPS* Karnofsky performance status, *RPA* recursive partition analysis, *SRS* stereotactic radiosurgery, *SRT* stereotactic radiotherapy, *WBRT* whole brain radiation therapy

^a^Nerologic function, gradeO as no symptoms, grade 1 as minor symptoms: fully active without assistance, grade2 as moderate symptomes; fully active but requires assistance, grade3 as moderate symptoms: less than fully active, requires assistance, grade4 as severe symptoms; totally inactive

^b^Thoracic stage are classified according to TNM classification of malignant tumors version 6 (UICC, Union for International Cancer Contorol version 6 edition) not evaluating M stage

The median age was 64 years (range: 22–86 years). There were 30 males and 31 females. Eleven patients were in the sync-oligometastases group with active primary lesions (thorax). On the other hand, 50 patients in the oligo-recurrence group had controlled primary lesions (thorax). The number of patients with KPS scores 70–80 and 90–100 were 5 and 56, respectively. As for histopathology, 6, 48, and seven patients had squamous cell carcinoma, adenocarcinoma, and other classified NSCLC, respectively. According to RPA class, a previously proposed and widely used prognostic factor for brain metastases [10], the 61 patients could be classified into two groups: RPA class I (*n* = 26) and RPA class II (*n* = 35). Because the current study included only oligometastases, the number of RPA class III patients was 0. Furthermore, Graded Prognostic Assessment (GPA, newly proposed prognostic factor of brain metastases) also could be classified into two groups: Intermediate Prognosis Group (GPA score: 1.5–3.0, *n* = 50) and Favorable Prognosis Group (GPA score: 3.5–4.0, *n* = 11) [9]. The number of lesions was 1–2 in 54 patients and 3–5 in seven patients. As for the number of metastatic or recurrent lesions limited to the brain, 42 were solitary, and 19 were 2–4. The median maximum size of metastatic or recurrent brain tumors was 1.2 cm (range: 0.2–6.0 cm).

The treatment methods for brain metastases or recurrences were SRS in 45 patients and SRT in 16 patients. The median prescription dose to PTV peripheral was 25 Gy (range: 10–36 Gy). External radiation therapy was used concomitantly in nine patients. Nineteen patients underwent chemotherapy. Neurologic function was based on Professor Aoyama’s standard reported in JAMA [11].

In brief, patients with no symptoms were assigned a Grade of 0; patients with minor symptoms but who were fully active without assistance were Grade 1; those with moderate symptoms who were fully active but required assistance were Grade 2; those with moderate symptoms but who were less than fully active and required assistance were Grade 3; and patients with severe symptoms who were totally inactive were Grade 4. In the current study, 40 patients had Grade 0, 12 had Grade 1, 9 had Grade 2, and no patients had Grade 3 or 4. As for NSCLC staging excluding metastatic or recurrent lesions (thoracic stage), 27 patients had stage I or II, and 34 had stage III disease. Forty-three patients were treated with surgery for thoracic lesions, and 18 patients were treated with concurrent chemoradiotherapy or radiation therapy alone.

### Survival

**Fig. 1:**
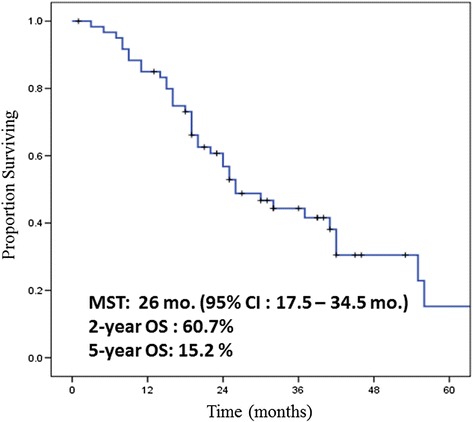
Overall survival (OS) of all patients. The median OS is 26 months (95 % CI: 17.5–34.5 months). The 2-year OS and 5-year OS are 60.7 and 15.2 %, respectively. These results are favorable although all patients having metastases or recurrences

The median overall survival of all 61 patients reached 26 months (95 % CI: 17.5–34.5 months). The 2-year and 5-year OS rates were 60.7 and 15.2 %, respectively (Fig. 1).

**Fig. 2:**
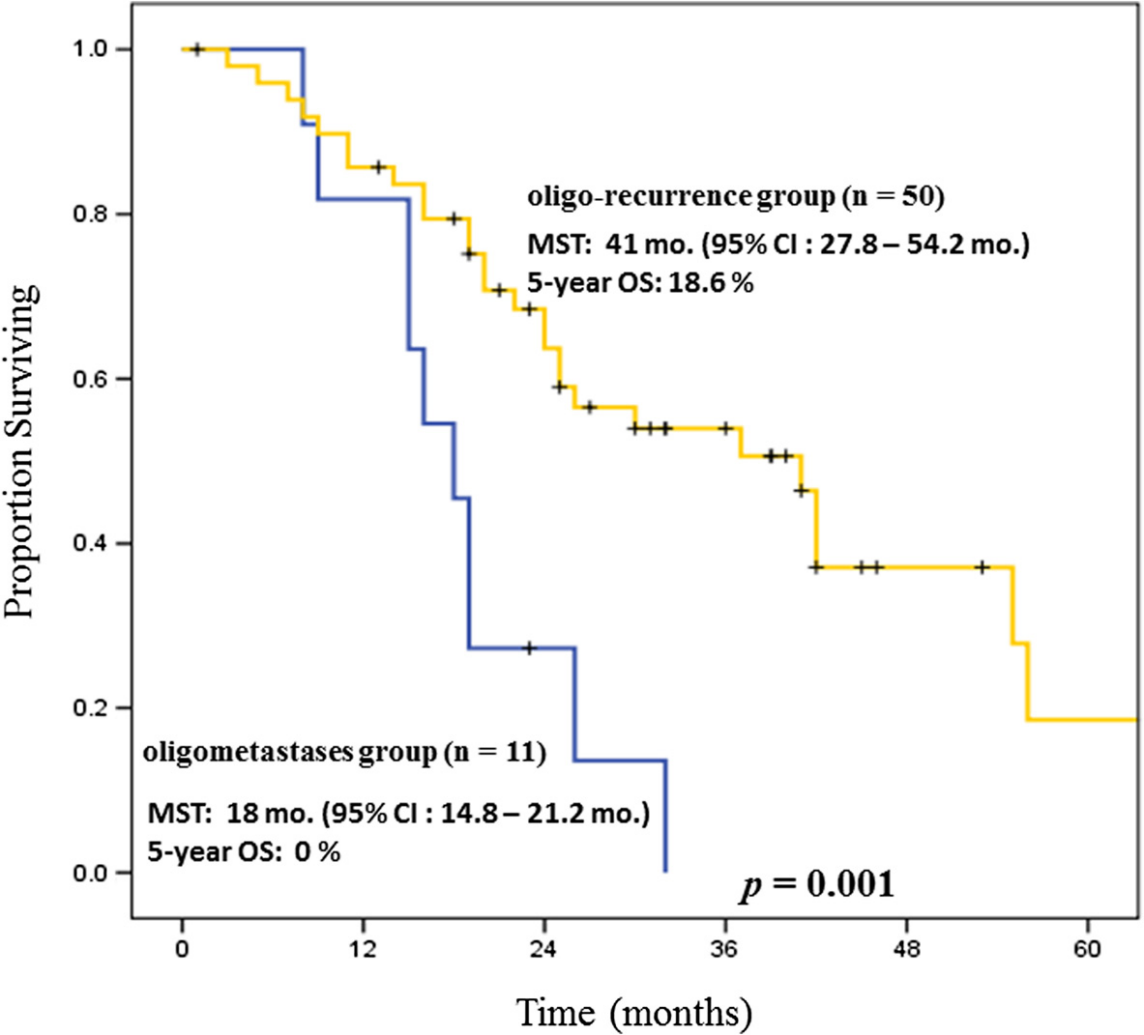
Overall survival (OS) stratified by oligostatus. The oligo-recurrence group has a significantly better OS than the oligometastases group. The oligo-recurrence group has an MST of 41 months independent of driver oncogenes

The results of univariate analysis of prognostic factors for OS are shown in Table 3. Oligostatus (*p* = 0.001) and the number of metastatic or recurrent lesions (*p* = 0.031) were both significant prognostic factors. Oligostatus was a powerful factor (Fig. 2); the sync-oligometastases group achieved a median OS of 18 months (95 % CI: 14.8–21.2 months) and a 5-year OS of 0 %. On the other hand, the oligo-recurrence group achieved a median OS of 41 months (95 % CI: 27.8–54.2 months) and a 5-year OS of 18.6 %.

**Table 3:** Univariate Analysis of Overall Survival

Variable	No.	Survival time, median (ranqe), mo	*p* value
Age, median (range), y			
<65	30	26 (17.2–34.8)	0.962
≥65	31	26 (8.3–43.8)	
Sex			
Male	30	32 (12.9–51.1)	0.489
Female	31	24 (13.8–34.2)	
No. of metastatic/recurrent lesions			
1–2	54	32 (18.0–46.0)	0.031
3–5	7	18 (10.3–25.7)	
Oligostatus			
Oligometastases (primary active)	11	18 (14.8–21.2)	0.001
Oligo-recurrence (primary controlled)	50	41 (27.8–54.2)	
Histological status			
Squamous cell carcinoma	6	32 (18.4–45.6)	0.233
Adenocarcinoma	48	19 (7.0–31.0)	
Others	7	19 (8.7–29.3)	
KPS score			
70–80	5	24 (9.9–38.1)	0.169
90–100	56	32 (19.2–44.8)	
Interval to brain recurrence, months			
<12	29	19 (14.9–23.2)	0.081
≥12	32	41 (34.6–47.4)	
No. of brain metastases			
Single	42	26 (11.3–40.7)	0.817
2–4	19	26 (16.5–35.5)	
RPA			
Class I	26	30 (8.6–51.4)	0.319
Class II	35	25 (10.6–39.4)	
GPA			
Intermediate Prognosis Group (1.5–3.0)	50	26 (16.9–35.1)	0.577
Favorable Prognosis Group (3.5–4.0)	11	25 (0.0–51.8)	
Neurologic function ^a^			
Grade 0–1	40	26 (11.1–40.9)	0.945
Grade 2–4	21	26 (16.5–35.5)	
Treatment method for brain tumor			
SRS	45	26 (17.3–34.7)	0.792
SRT	16	25 (1.7–48.3)	
WBRT			
Yes	9	25 (11.1–38.9)	0.774
No	52	26 (12.0–40.0)	
Thoracic stage ^b^			
I–II	27	25 (16.8–33.2)	0.199
III	34	37 (20.1–53.9)	
Treatment method for thoracic lesions			
Surgery	43	30 (20.2–39.8)	0.94
Radiation therapy	18	25 (3.2–46.8)	
Chemotherapy			
Yes	19	26 (24.1–27.9)	0.975
No	42	30 (9.0–51.0)	

Abbreviations: *KPS* Karnofsky performance status, *RPA* recursive partition analysis, *GPR* graded prognostic assessment, *SRS* stereotactic radiosurgery, *SRT* stereotactic radiotherapy, *WBRT* whole brain radiation therapy

^a^Neurologic function, grade 0 as no symptoms; grade 1 as minor symptoms, fully active without assistance; grade 2 as moderate symptoms, fully active but requires assistance; grade 3 as moderate symptoms, less than fully active, requires assistance; grade 4 as severe symptoms, totally inactive

^b^Thoracic stage classified according to the TNM classification of malignant tumors version 6 (UICC, Union for International Cancer Control version 6 edition) not evaluating M stage

**Fig. 3:**
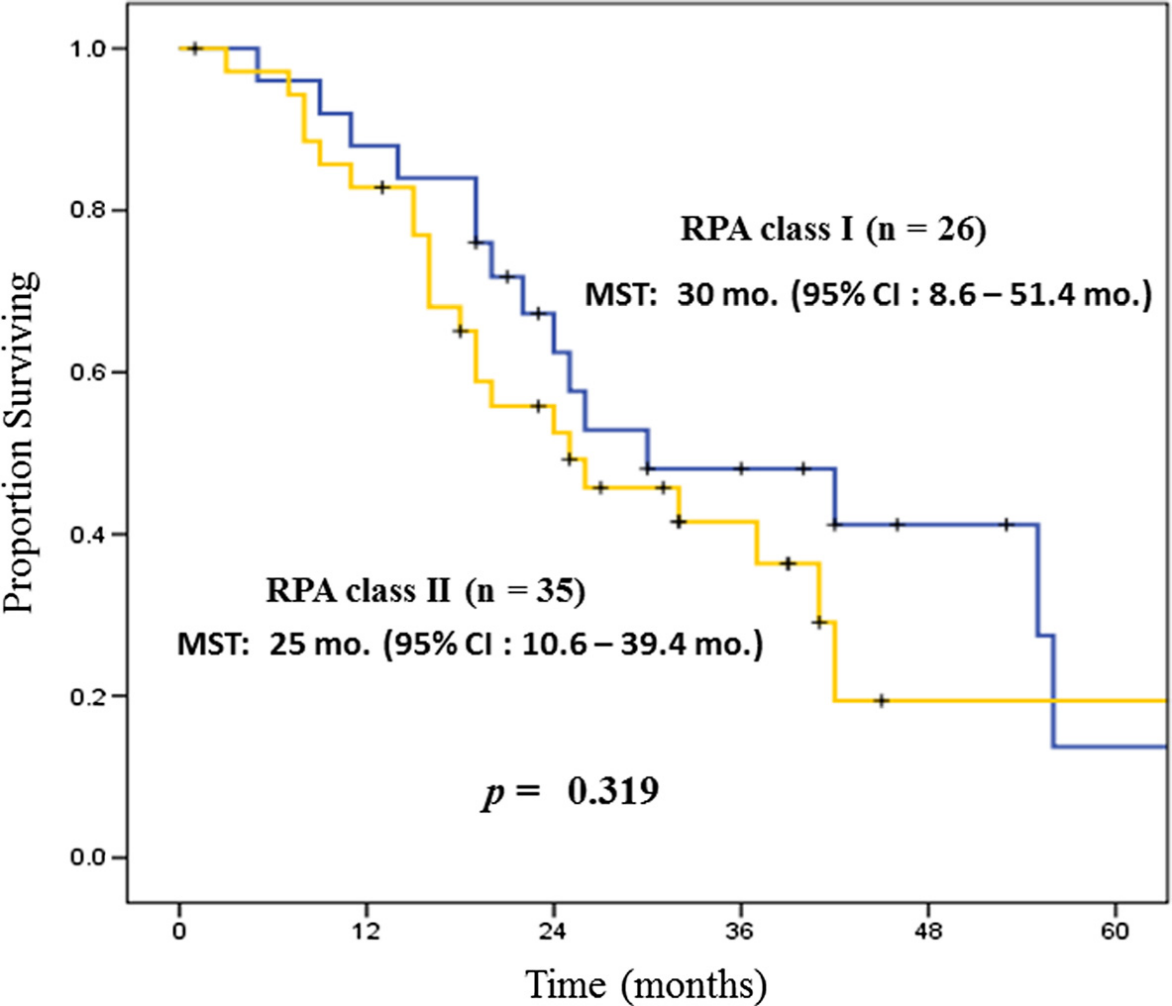
Overall survival (OS) stratified by RPA class. RPA is not a significant prognostic factor for brain metastases, despite its wide use for this purpose

However, RPA, the most frequently used standard for the prediction of prognosis of patients with brain metastases, achieved no significance for OS of NSCLC oligometastases. RPA class I achieved a median OS of 30 months (95 % CI: 8.6–51.4 months), and RPA class II achieved a median OS of 25 months (95 % CI: 10.6–39.4 months) (*p* = 0.319). These results were almost identical (Fig. 3). Furthermore, newly proposed prognostic classification, GPA also achieved no significance for OS of NSCLC oligometastases. Intermediate Prognosis Group of GPA scoring 1.5–3.0 achieved a median OS of 26 months (95 % CI: 16.9–35.1 months), and Favorable Prognostic Group of GPA scoring 3.5–4.0 achieved a median OS 25 months (95 % CI: 0.0–51.8 months) (*p* = 0.577) These results were almost identical (Table 3). This was a very important finding of the current analysis. In addition, a multivariate analysis was performed using the factors that were found to be significant on univariate analysis (oligostatus, the number of metastatic or recurrent lesions), those that showed a nonsignificant trend toward significance (*p* < 0.25), and clinically important factors (RPA, histopathology, KPS score, interval to brain recurrence (DFI), thoracic stage). The results of multivariate analysis are shown in Table 4. Oligo-recurrence was extracted as the only independent prognostic factor (hazard ratio: 0.253; 95 % CI: 0.082–0.043) (*p* = 0.025).

**Table 4:** Multivariate Analysis of Overall Survival

Variable	Hazard ratio (95 % Cl)	*p* value
No. of metastatic/recurrent lesions (1–2)	0.453 (0.152–1.240)	0.155
Oligo-recurrence group	0.253 (0.082–0.843)	0.035
Adenocarcinoma	0.496 (0.211–1.164)	0.107
KPS score (90–100)	0.330 (0.120–1.066)	0.064
Interval to brain recurrence, ≥12 months	1.158 (0.509–2.636)	0.726
RPA (class I)	1.319 (0.556–3.071)	0.521
Thoracic stage ^a^ (I–II)	1.544 (0.777–3.066)	0.215

Abbreviations: *KPS* Karnofsky performance status, *RPA* recursive partition analysis

^a^Thoracic stage is classified according to the TNM classification of malignant tumors version 6 (UICC, Union for International Cancer Control version 6 edition) not evaluating M stage

The median relapse-free survival reached 10 months (95 % CI: 7.32–12.7 months), and the 2-year and the 5-year RFS rates were 30.3 and 6.6 %, respectively.

### Local control

Cranial-LC for all patients achieved a median of 30 months (95%CI: 18.1–41.8 months), and 2-year Cranial-LC and 5-year Cranial-LC rates were 68.1 and 10.7 %, respectively. Univariate analysis of Cranial-LC was performed. There were no significant factors for prognosis in Cranial-LC. A Cox proportional hazards model multivariate analysis of Cranial-LC was performed using factors with a nonsignificant trend toward significance and clinically important factors (oligostatus, the number of brain metastases or recurrences, and whole brain irradiation). Multivariate analysis also found no significant factors.

**Fig. 4:**
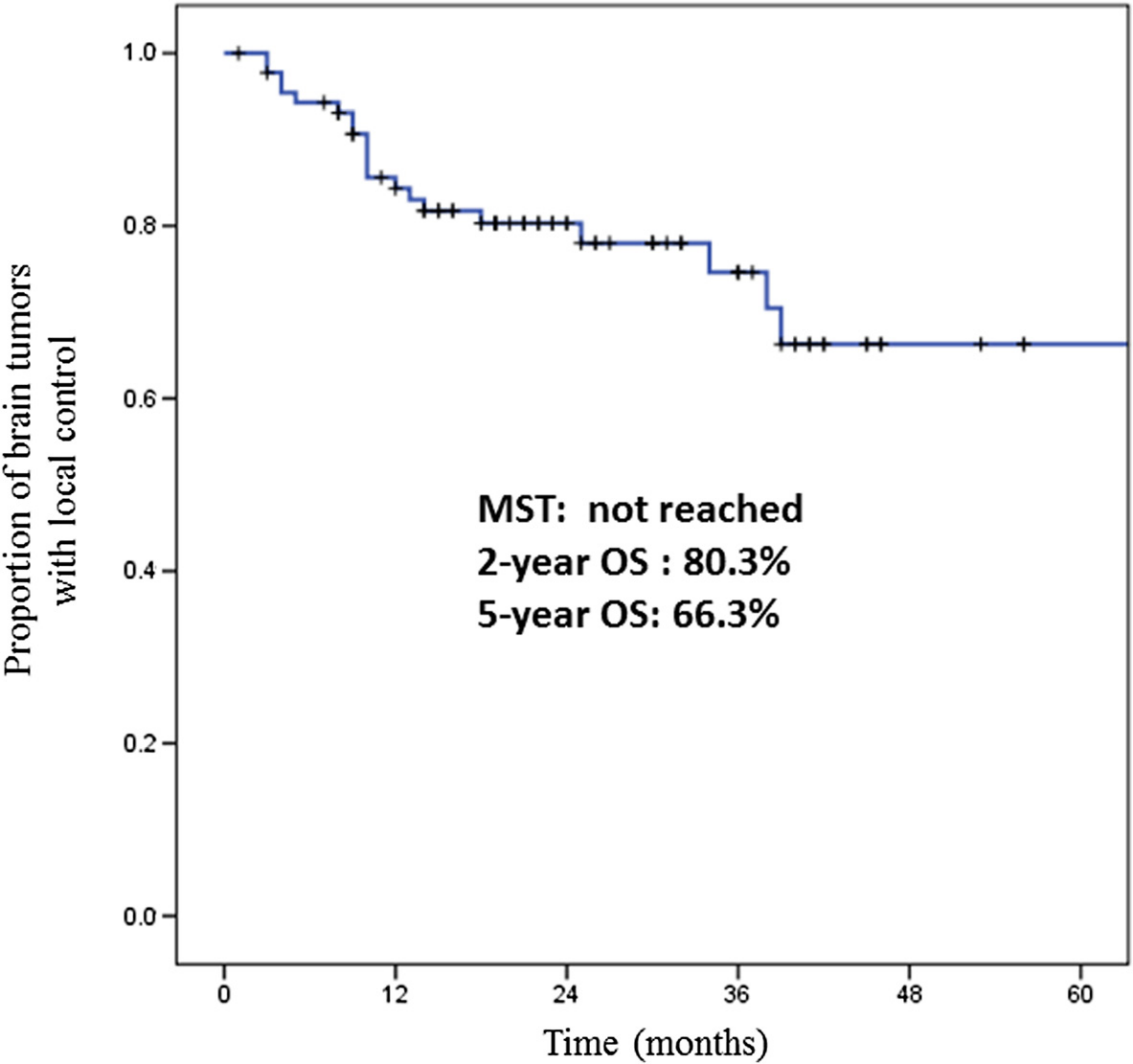
Brain local control rate (Brain-LC) of all patients. The 2-year and 5-year Brain-LC rates are 80.3 and 66.3 %, respectively. These results are comparable to previous studies

Brain-LC of all patients achieved a median LC rate of not reached, and 2-year Brain-LC and 5-year Brain-LC rates were 80.3 and 66.3 %, respectively (Fig. 4). The results of univariate analysis of Brain-LC are described in Table 5. Histopathology (*p* = 0.017) and the maximum tumor diameter (≥3 cm) (*p* = 0.002) were significant prognostic factors. However, the relationship between histopathology and the maximum tumor diameter (<3 cm) was evaluated, and there was a correlation between histopathology and the maximum tumor diameter. Nine of 11 tumors with squamous cell carcinoma had a maximum tumor diameter <3 cm (81.8 %). On the other hand, 68 of 69 adenocarcinomas had a maximum tumor diameter <3 cm (98.6 %), and 6 of 9 NSCLCs with other histology had a maximum tumor diameter < 3 cm (66.7 %). Thus, adenocarcinoma tumors had a tendency to be smaller than squamous cell carcinoma or other NSCLC tumors. Histopathology was therefore excluded from the multivariate analysis, and maximum tumor diameter was included. The results of the Cox proportional hazards model multivariate analysis are shown in Table 6. The maximum tumor diameter showed a nonsignificant trend toward significance (the maximum tumor diameter was ≥3 cm; hazard ratio 3.81; 95 % CI: 0.95–15.3) (*p* = 0.059).

**Table 5:** Univariate Analysis of Local Control of Brain Tumor

Variable	No. of tumors	2-year control rate, months	*p* value
Histological status			
Squamous cell carcinoma	11	53.7 % (11 months)	0.017
Adenocarcinoma	69	85.50 %	
Others	9	55.60 %	
Size of brain tumor			
<3 cm	83	82.80 %	0.002
≥3 cm	6	44.40 %	
Treatment method for brain tumor			
SRS	66	84.90 %	0.098
SRT	23	60.0 % (23 months)	
WBRT			
Yes	13	84.60 %	0.701
No	76	81.30 %	
Chemotherapy			
Yes	29	79.40 %	0.725
No	60	73.90 %	

Abbreviations: *SRS* stereotactic radiosurgery, *SRT* stereotactic radiotherapy, *WBRT* whole brain radiation therapy

**Table 6:** Multivariate Analysis of Local Control of Brain Tumc

Variable	Hazard ratio (95 % Cl)	*p* value
Size of brain tumor (≥3 cm)	3.81 (0.95–15.3)	0.057
SRS	0.632 (0.425–4.10)	0.828

Abbreviations: *SRS* stereotactic radiosurgery

Thoracic-LC of all patients achieved a median LC rate of not reached, and 2-year Thoracic-LC and 5-year Thoracic-LC rates were 80.5 and 64.3 %, respectively. Univariate analysis of Thoracic-LC was performed. Only oligostatus (*p* = 0.035) and use of systemic chemotherapy (*p* = 0.022) were significant prognostic factors. However, the relationship between oligostatus and systemic chemotherapy was investigated, and a significant correlation between these two factors was found. In the oligo-recurrence group, 12 of 50 patients (24 %) underwent systemic chemotherapy. On the other hand, in the sync-oligometastases group, 7 of 11 patients (63.6 %) underwent systemic chemotherapy. Thus, systemic chemotherapy was excluded from the multivariate analysis. The multivariate analysis thus included only oligostatus and one clinically important factor (thoracic stage). Oligostatus was not an independent prognostic factor (hazard ratio for oligo-recurrence: 0.405; 95 % CI: 0.121–1.353) (*p* = 0.142).

## Discussion

Stage IV or recurrent stage IV lung cancer patients were considered to be end-stage cancer patients until the early 2000s. However, rapid progress was achieved in clinical molecular targeted drugs of lung cancer in the mid-2010s. It appears that some types of oncogenes regulate lung cancer progression or suppression, such as EGFR or ALK [12–14]. Based on these results, EGFR-TKIs and ALK-inhibitors have been identified and manufactured after FDA (US), EMA (Europe), and PMDA (Japan) approval.

However, these patients are all oncogene-driven lung cancer patients. Patients with lung cancer unrelated to driver oncogene mutations still suffer from poor QOL and low survival rates.

On the other hand, in the current study, oligo-recurrent patients with NSCLC treated by SRS or SRT achieved longer survival not depending on driver oncogenes. The MST of OS in the oligo-recurrence group reached 41 months (95 % CI: 27.8–54.2 months). Moreover, these patients could achieve a “cure”. The 2-year and 5-year OS rates were 60.7 and 15.2 %, respectively, for the oligo-recurrence group. Molecular-targeted drugs could achieve longer survival for specific NSCLC patients. However, these drugs could not achieve a “cure” due to the acquired resistance of tumor cells. This point is very important.

Ashworth et al. recently reported from their meta-analysis that the most important prognostic factor was metachronous oligometastases in patients with NSCLC oligometastases [15]. However, these results must be evaluated very cautiously. The use of metachronous oligometastases is not appropriate in the area of oligometastases. Metachronous oligometastases brought similar good results to oligo-recurrence. However, metachronous oligometastases include concomitant relapse of primary and metastatic lesions, although oligo-recurrence excludes this type of relapse.

Concomitant relapse of primary and metastatic lesions is a state similar to that of sync oligometastases, rather than one resembling oligo-recurrence. Thus, for this type of relapse we cannot achieve good OS while maintaining good QOL. Second, in the medical scientific literature, the concept of oligo-recurrence was proposed before that of metachronous oligometastases. Thus, oligo-recurrence was the original concept related to oligometastases with controlled primary lesions. Accordingly, the meta-analysis of Ashworth should be revised using the appropriate key term of oligo-recurrence.

The multivariate analysis using a Cox proportional hazards model in the current study concluded that oligo-recurrence was the best prognostic factor in brain-only oligometastases in patients with NSCLC (hazard ratio: 0.253; 95 % CI: 0.082–0.043) (*p* = 0.025) (Table 4).

The major contribution of this analysis thus goes beyond the above-described finding that oligo-recurrence was the only independent prognostic factor. Until now, the standard prognostic factor for brain metastases has been RPA class or GPA [9, 10]. However, Fig. 3 and Table 2 shows that neither RPA nor GPA were significant prognostic factors for oligometastases of NSCLC (RPA, *p* = 0.319) (GPA, *p* = 0.577). The multivariate analysis also confirmed that RPA was not a prognostic factor for oligometastases of NSCLC (hazard ratio: 1.31; 95 % CI: 0.557–3.05) (*p* = 0.54).

The 2-year and 5-year Cranial-LC rates were 68.1 and 10.7 %, respectively. The median control time of Cranial-LC was 30 months (95 % CI: 18.1–41.8 months). Increasing Cranial-LC requires whole brain radiation therapy combined with SRS or SRT. However, neurocognitive function decreases when patients undergo whole brain radiation therapy [16]. Thus, recently, when intracranial relapse occurred, we elected to perform SRS or SRT repeatedly whenever possible. In the current study, therefore, only nine patients underwent whole brain radiation therapy. Thus, the results of Cranial-LC are not good.

The Brain-LC rates of all patients achieved the median LC rate of not reached, and the 2-year Brain-LC and 5-year Brain-LC rates were 80.3 and 66.3 %, respectively. These results were almost the same as the findings reported previously [11]. The present study thus confirms that SRS or SRT for small brain metastases achieves good treatment results as an alternative to surgery (log-rank test, maximum tumor diameter brain tumors <3 cm achieve 2-year Brain-LC of 82.8 %, *p* = 0.002; multivariate analysis, ≥3 cm, hazard ratio 3.81 (95 % CI: 0.95–15.3), *p* = 0.059 (marginally significant correlation of Brain-LC)).

Thoracic-LC findings in the current study were as follows. The 2-year Thoracic-LC and 5-year Thoracic-LC rates were 80.5 and 64.3 %, respectively. These results were reasonable in the current study setting.

There are some limitations in the current study. First, this is retrospective study. The number of registered patients is limited to 61 subjects. There are some difference in the treatment methods even among six high volume institutions.

## Conclusions

In conclusion, oligo-recurrence can only predict a favorable prognosis of brain-only oligometastases in patients with non-small cell lung cancer treated with SRS or SRT. To the best of our knowledge, this is the first clinical demonstration that oligo-recurrence is the most important favorable prognostic factor for oligometastases in NSCLC. We are currently conducting a prospective study of oligometastases to confirm that oligo-recurrence is the most important favorable prognostic factor in NSCLC and other cancers.

## Abbreviations

Brain-LC: Local control of brain metastases
Cranial-LC: Cranial local control
DFI: Interval to initial brain recurrence
KPS: Karnofsky performance status
NSCLC: Non-small cell lung cancer
OS: Overall survival
RFA: Radiofrequency ablation
RFS: Relapse-free survival
RPA: Recursive partition analysis
SBRT: Stereotactic body radiotherapy
SRS: Stereotactic radiosurgery
SRT: Stereotactic radiotherapy
Thoracic-LC: Local control of thoracic lesions
